# A Microfluidic Channel Method for Rapid Drug-Susceptibility Testing of *Pseudomonas aeruginosa*

**DOI:** 10.1371/journal.pone.0148797

**Published:** 2016-02-12

**Authors:** Yoshimi Matsumoto, Shouichi Sakakihara, Andrey Grushnikov, Kazuma Kikuchi, Hiroyuki Noji, Akihito Yamaguchi, Ryota Iino, Yasushi Yagi, Kunihiko Nishino

**Affiliations:** 1 Institute of Scientific and Industrial Research, Osaka University, Osaka, Japan; 2 Department of Applied Chemistry, Graduate School of Engineering, University of Tokyo, Tokyo, Japan; 3 Okazaki Institute for Integrative Bioscience and Institute for Molecular Science, National Institutes of Natural Sciences, Okazaki, Japan; 4 The Graduate University for Advanced Studies (SOKENDAI), Kanagawa, Japan; Purdue University, UNITED STATES

## Abstract

The recent global increase in the prevalence of antibiotic-resistant bacteria and lack of development of new therapeutic agents emphasize the importance of selecting appropriate antimicrobials for the treatment of infections. However, to date, the development of completely accelerated drug susceptibility testing methods has not been achieved despite the availability of a rapid identification method. We proposed an innovative rapid method for drug susceptibility testing for *Pseudomonas aeruginosa* that provides results within 3 h. The drug susceptibility testing microfluidic (DSTM) device was prepared using soft lithography. It consisted of five sets of four microfluidic channels sharing one inlet slot, and the four channels are gathered in a small area, permitting simultaneous microscopic observation. Antimicrobials were pre-introduced into each channel and dried before use. Bacterial suspensions in cation-adjusted Mueller–Hinton broth were introduced from the inlet slot and incubated for 3 h. Susceptibilities were microscopically evaluated on the basis of differences in cell numbers and shapes between drug-treated and control cells, using dedicated software. The results of 101 clinically isolated strains of *P*. *aeruginosa* obtained using the DSTM method strongly correlated with results obtained using the ordinary microbroth dilution method. Ciprofloxacin, meropenem, ceftazidime, and piperacillin caused elongation in susceptible cells, while meropenem also induced spheroplast and bulge formation. Morphological observation could alternatively be used to determine the susceptibility of *P*. *aeruginosa* to these drugs, although amikacin had little effect on cell shape. The rapid determination of bacterial drug susceptibility using the DSTM method could also be applicable to other pathogenic species, and it could easily be introduced into clinical laboratories without the need for expensive instrumentation.

## Introduction

Bacterial antibiotic resistance is an alarming issue, affecting veterinary and human health. There are various factors responsible for the emergence of resistance, including the inappropriate use of antibiotics and patient-related factors such as growing numbers of immunocompromised patients because of improvements in medical care and an increasingly aging population. *Pseudomonas aeruginosa* is one of the major causative agents of hospital-acquired infections, particularly among patients admitted to the intensive care unit. *P*. *aeruginosa* infections, particularly those in immunocompromised patients, often result in life-threatening disease [1, 2] and are untreatable because of resistance to multiple antimicrobial agents. The low permeability of the bacterial membrane facilitates its inherent resistance to many antibiotics and disinfectants.[3–5] Bacterial drug resistance can be amplified both quantitatively and qualitatively by the acquisition of additional drug resistance factors.[6, 7] The tendency of intrinsic resistance in *P*. *aeruginosa* makes it difficult to eradicate this opportunistic pathogen from hospital environments. Multidrug-resistant *P*. *aeruginosa* (MDRP) strains resistant to major antipseudomonal agents such as carbapenems, quinolones, and aminoglycosides, have recently become prevalent [8, 9] and have caused nosocomial outbreaks in Japan.[10, 11] Few antibiotics are available for the treatment of MDRP infections, and one of the few effective drugs against this pathogen, colistin [12] was only available until very recently in Japan. Moreover, new drug development is lacking.

The definitions of multidrug-resistance (MDR) in *P*. *aeruginosa* have not been unified globally. MDRP is defined as a strain that has acquired non-susceptibility to at least one agent in three or more categories of antimicrobials according to the Centre for Disease Control and Prevention and the European Center for Disease Prevention and Control. In Japan, the term MDRP indicates resistance to three different classes of antibiotics, amikacin (AMK, aminoglycoside), ciprofloxacin (CIP, quinolone) and imipenem (IMP, carbapenem). MDRP possesses complex drug resistance mechanisms,[13–16] and several barriers are known to attenuate the activity of β-lactams. First, the bacterium’s relatively impermeable outer membrane decreases the access of most hydrophilic β-lactams to their target proteins in the periplasm. The outer membrane porin protein OprD allows the entry of carbapenems, [17, 18] and *oprD* downregulation is the most important mechanism of resistance to this class of drugs.[19–21] Second, chromosomal and transferable β-lactamases inactivate β-lactams in the periplasm. Among various β-lactamases identified in *P*. *aeruginosa*, metallo-β-lactamases (MBLs, class B),[22] in particular, can hydrolyze virtually all classes of β-lactams excluding aztreonam.[23–25] Finally, multidrug efflux pumps, particularly resistance-nodulation-cell division (RND) family pumps, can decrease the sensitivity of *P*. *aeruginosa* to various toxic compounds [26, 27] and the action of RND pumps alone can confer MDR. Of these pumps, the MexAB-OprM and MexXY-OprM efflux systems contribute significantly to drug resistance.[28, 29] MexB is known to export various antimicrobial agents including quinolones and a number of β-lactams excluding IPM,[18, 27] whereas MexY is known to export aminoglycosides and quinolones.[27] In addition to the aforementioned mechanisms, resistance to quinolones can occur because of mutations in DNA gyrase and/or topoisomerase IV.[30] The expression of enzymes that modify aminoglycosides or their target sites on the ribosome [31] could also confer aminoglycoside resistance. Increased efflux might additively or synergistically affect these mechanisms to further enhance resistance.[32] The complicating influence of multiple factors affect the antibiotic susceptibility of *P*. *aeruginosa*, which makes it difficult to evaluate the actual susceptibilities of the bacterium to drugs using rapid methods such as PCR and immunochromatography.

At the beginning of this study, we investigated the susceptibility of clinically isolated strains of *P*. *aeruginosa* and the correlation of susceptibility with several resistance factors. Although previous studies [5, 6, 33, 34] investigating the overexpression of efflux pump genes and decreased expression of *oprD* in some resistant *P*. *aeruginosa* strains suggested a role for pumps and porin in MDR, data relating to expression levels of these genes in sensitive strains were not enough. To evaluate the properties of efflux pump and porin genes as MDRP markers, it is important to examine the differences in the expression levels of resistance genes in large numbers of sensitive and resistant strains, simultaneously. We evaluated the expression levels of *mexB*, *mexY*, and *oprD* in resistant and sensitive strains using quantitative real-time RT-PCR. Fifty-four MDRP strains resistant to IPM, AMK, and CIP were compared with 26 sensitive strains and 21 resistant strains, other than MDRP. In addition to these three genes, the productive ability of MBL and 6’-N-aminoglycoside acetyltransferase-Iae [10, 35] was detected using immunochromatography. The acquisition of multiple resistance factors was frequently detected in MDRP strains, particularly those producing MBL. However, this was also observed in some sensitive strains (Table A in S1 File). Based on the weak correlation between the expression levels of the resistance genes and actual resistance levels in these strains, we found it difficult to estimate the resistance levels of strains using several acquired resistance factors and/or by the expression levels of several intrinsic genes responsible for drug resistance. Similar results were previously obtained by El Amin et al. [36].

Given this background, rapid determination of drug susceptibility in order to select effective drugs for each patient is promptly required. However, the development of drug susceptibility testing methods has not yet been completely accelerated, in contrast to identification methods,. The latter have become extremely rapid via the introduction of MALDI-TOF MASS.[37, 38] It is important to consider why drug susceptibility testing remains time consuming. Fully automated systems such as WalkAway (Beckman Coulter), BD phoenix (Becton Dickinson and Company), and Vitek 2 (bioMérieux Industry) are still not rapid enough with respect to susceptibility testing. The biggest reason for this is that we use optical density or colorimetric methods to detect bacterial growth, which require control bacteria to become visible after the incubation of intact cells with drugs. Meanwhile, we have attempted to apply nanotechnology to microbiological experiments,[39–41] and have developed a new method to determine efflux pump activity in *Escherichia coli*. [39, 40] Using microfluidic channels with different shapes, we attempted to determine the susceptibilities of *P*. *aeruginosa* isolates to antimicrobial agents in a period of ≤3 h. In contrast to other rapid methods recently published using microscopy,[42–52] our protocol is extremely simple with no expensive instrumentation needed. The method does not use continual time lapse study, therefore a technician can analyze susceptibility of several strains at the same time. In this study, we proposed a new simple, rapid, and cheap drug susceptibility testing method comparable to the microbroth dilution method according to the Clinical Laboratory Standards Institute (CLSI, USA) guidelines.[53]

## Materials and Methods

### Bacterial strains

*P*. *aeruginosa* ATCC27853 was used as a standard strain for drug susceptibility testing. *Escherichia coli* ATCC25922 was also used in the time-lapse study. Based on the breakpoints recommended by CLSI in 2007, MDRP was defined as a strain that grows in the presence of 16 mg/L AMK, 2 mg/L CIP and 8 mg/L IPM. Strains isolated from clinical specimens in the laboratories of BML, Inc. (Kawagoe, Japan) in 2007 were divided into three groups as follows: MDRP, resistant to all three compounds; RP, resistant to one or two of the three agents and sensitive *P*. *aeruginosa* (SP), sensitive to all three agents. Strains isolated from the same hospital and having similar sensitivity patterns were excluded from the list. We tested 55 MDRP, 21 RP, and 26 SP strains in total.

### Antimicrobial agents and chemicals

Antimicrobials included AMK (Nichi-Iko Pharmaceutical Co., Ltd. Toyama, Japan), CIP (Meiji Seika Kaisha, Ltd., Tokyo, Japan), meropenem (MPM; Meiji Seika Kaisha, Ltd.), ceftazidime (CAZ; Sawai Pharmaceutical Co., Ltd, Osaka, Japan), and piperacillin (PPC; TOYAMA CHEMICAL CO., LTD, Tokyo, Japan).

### Preparation of microfluidic channels

**Fig 1:**
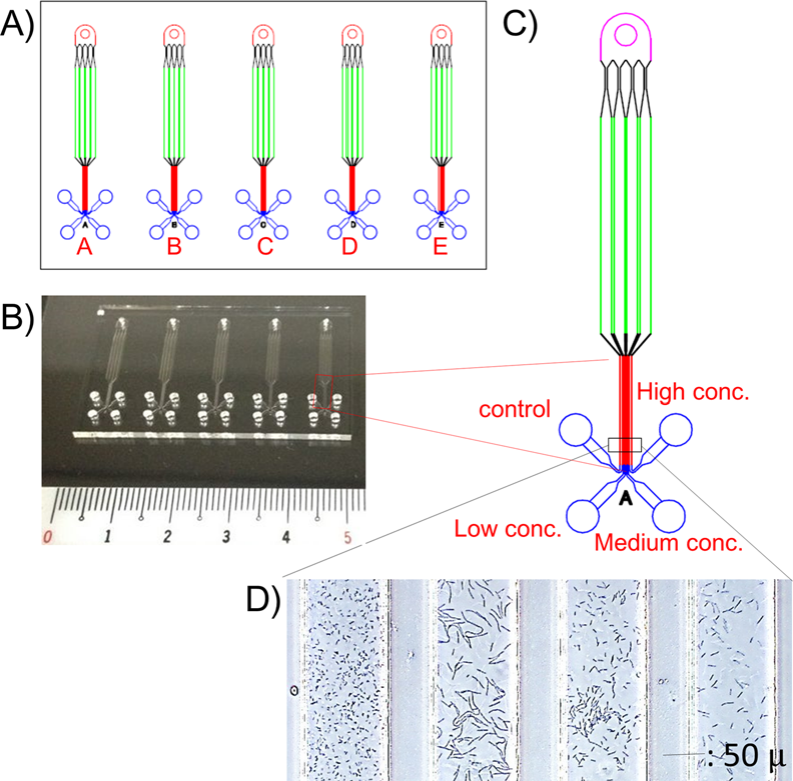
The structure of the drug susceptibility testing microfluidic (DSTM) device for MIC determination of five drugs. A) Design of the DSTM device used in the study. B) Actual image of the DSTM device. C) Precise structure of one set of fluids. D) Microscopic image of *Pseudomonas aeruginosa* grown in the presence of piperacillin (0, 4, 8, and 16 mg/L) for 3 h. Width of each fluid is 100 μm.

The drug susceptibility testing microfluidic (DSTM) device was prepared as follows. Microfluidic channels (width, 100 μm; height, 50 μm; length 3–5 mm in the observation area) were fabricated in polydimethylsiloxane (PDMS; Silpot184, Dow Corning Toray Co., Ltd., Tokyo, Japan), on a cover glass (Matsunami Glass Ind., Ltd., Osaka, Japan) using a conventional soft lithography method.[39, 54] We made DSTM devices containing five sets of four channels (Fig 1), allowing three different concentrations of five antimicrobials to be tested. Four channels shared an inlet hole, and they were gathered in the observation area. We selected five antimicrobials for treatment of *P*. *aeruginosa* as follows: AMK (4, 8, and 16 mg/L), CIP (1, 2, and 4 mg/L), MPM (1, 2, and 4 mg/L), CAZ (4, 8, and 16 mg/L), and PPC (4, 8, and 16 mg/L). Antimicrobials were dissolved in water, pre-introduced to each microfluidic channel from the outlet hole, and freeze-dried before use.

### The cell analyzer: software for the DSTM image analysis

**Fig 2:**
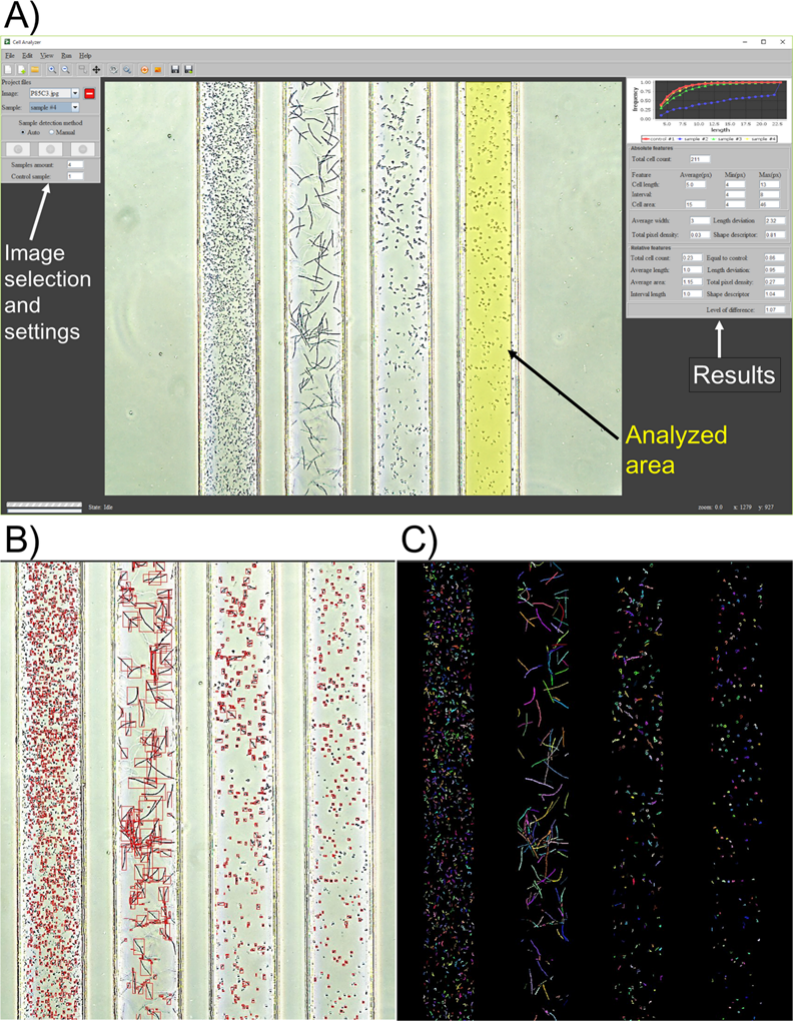
The cell analyzer for analysis of images using the DSTM method. A) Graphical interface of the software. Appropriateness of analyzed area can be checked and settled manually when they are not adequate. Calculated data in an Excel form were also available. B) Analyzed image to check bacterial cell counts. C) Image to confirm partially-overlapped long cells are counted separately.

To avoid human error in susceptibility judgement in the DSTM method, we prepared software (Fig 2) for the DSTM image analysis in order to count cell numbers, dependent on cell size. The cell analyzer can automatically analyze the DSTM images in a folder one by one. It detects fluid borders, defines the area for calculation on each fluid, and calculates several features, such as total amount of cells, average cell length, average cell area, and pixel density in comparison with those of control parameters. Standard deviations of cell length were also calculated.

### Determination of MICs

Determination of MICs using the DSTM device was conducted as follows. Bacterial cells grown overnight on Heart infusion agar (Becton, Dickinson and Company, Franklin Lakes, NJ, USA) were suspended in cation-adjusted Mueller–Hinton broth (OD = 0.07–0.13), and introduced into the DSTM device using a micropipette. Air introduced after the bacterial suspension was added to the DSTM device from the shared inlet hole was able to separate each channel completely. DSTM devices were incubated under humid condition at 37°C for up to 3 h. Results were evaluated microscopically in comparison with the control. A MT4210L phase contrast microscope with a 10-fold objective lens (MEIJI TECHNO, Saitama, Japan) and MIR-MDCE-5C USB2.0 digital camera (Bio Medical Science, Tokyo, Japan) were used. The images were processed using the aforementioned software.

The standard MICs of the used strains were determined by the ordinary microbroth dilution method[53] using a “Dry plate Eiken (DPD2)” (EIKEN Chemical Co., Ltd, Tokyo, Japan).

## Results

### Average growth of *P*. *aeruginosa* in the DSTM device

**Fig 3:**
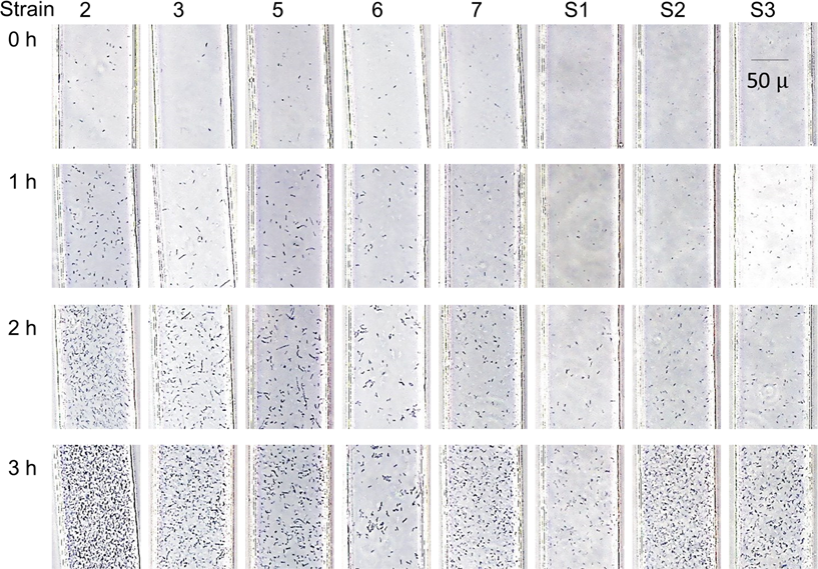
Growth of eight strains of *Pseudomonas aeruginosa* in the DSTM device over time. Suspensions of McFarland 0.4–0.5 were used. Strain 2 grew too rapidly to analyze images at 3 h using the software, although it was possible to judge this visually. The images of strains 2, 3, 5, and 7 at 2 h were useful for image analysis, and all images at 3 h except for strain 2 were analyzable using the software.

*P*. *aeruginosa* generally has a slow growth rate in comparison with species from the *Enterobacteriaceae*. *P*. *aeruginosa* is aerobic, and its growth is predominantly enhanced by aeration. Although PDMS is air-permeable, it is not sufficient to enhance the growth of *P*. *aeruginosa*, and the bacterium grew slowly in the fluids because of the lack of oxygen. We found that the presence of outlet holes near the observation area could increase the oxygen supply and accelerate the growth rate of *P*. *aeruginosa* in DSTM devices. Time-lapse analysis of the growth of eight *P*. *aeruginosa* strains was performed in the DSTM device with no antimicrobial agents (Fig 3). When we used bacterial suspensions of McFarland 0.4–0.5, one strain (No.2) grew rapidly, whereas two strains (No.6 and S1) grew slowly. Despite the variable growth rate of *P*. *aeruginosa* strains, 3 h of incubation was generally sufficient for susceptibility determination in these strains. Of 102 strains, 3 h of incubation was insufficient for susceptibility determination in only one strain (strain S9 missing in Table B in S1 File).

### Optimization of inoculum size for the DSTM method

**Fig 4:**
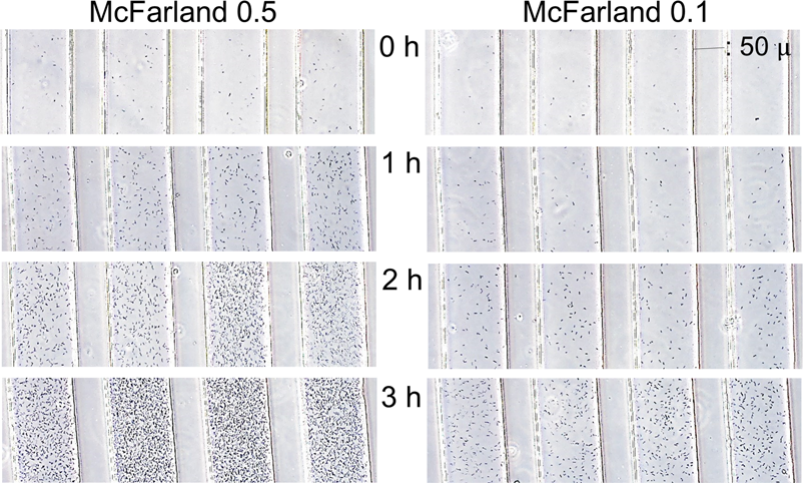
Effect of the inoculum size on the growth of *Pseudomonas aeruginosa* strain 2 in the DSTM device. Cell suspension with an OD of 0.122 was used as McFarland 0.5. McFarland 0.1 was suitable for analysis using the software at 3 h in this strain although images from McFarland 0.5 at 3 h resulted in growth too dense to be detected using the software.

In the DSTM device, the width of channels in the observation area is 100 μm, the length of channels in the monitor image is approximately 700 μm, and the depth of channels is 50 μm. Therefore, the volume of the bacterial suspension in one channel in one image is approximately 3.5 × 10^−6^ mL. When we use a bacterial suspension containing 1 × 10^6^ cells/mL, this means that there are only 3–4 cells in one channel in one image. Consequently, there were too few cells to detect them without much effort. When we used a bacterial suspension of McFarland 0.5 (approximately 1.5 × 10^8^ cells/mL), there was approximately 500 cells in one channel in one image, and it was easy to see modest number of cells at a focused depth. The inoculum sizes of McFarland 0.1 and 0.5 were compared in time-lapse analysis (Fig 4) using strain No. 2, which grew rapidly (see Fig 3). When McFarland 0.5 was used, excessive growth was obtained in this strain, and McFarland 0.1 was useful at 3 h. The lower inoculum size seemed better when we used this software. Finally, we decided to use the inoculum cell density of McFarland 0.2–0.3 for susceptibility testing of *P*. *aeruginosa* using the DSTM method. Using this condition, overgrowth-derived misjudgments using the software occurred in 2 of 102 strains tested, although these 2 strains could be judged visually. Taking pictures at 2 h incubation was useful for rapidly growing strains, and it is possible to wait for >3 h, if 3 h incubation was not enough for slowly growing strains.

### Morphological changes of *P*. *aeruginosa* in the DSTM device

**Fig 5:**
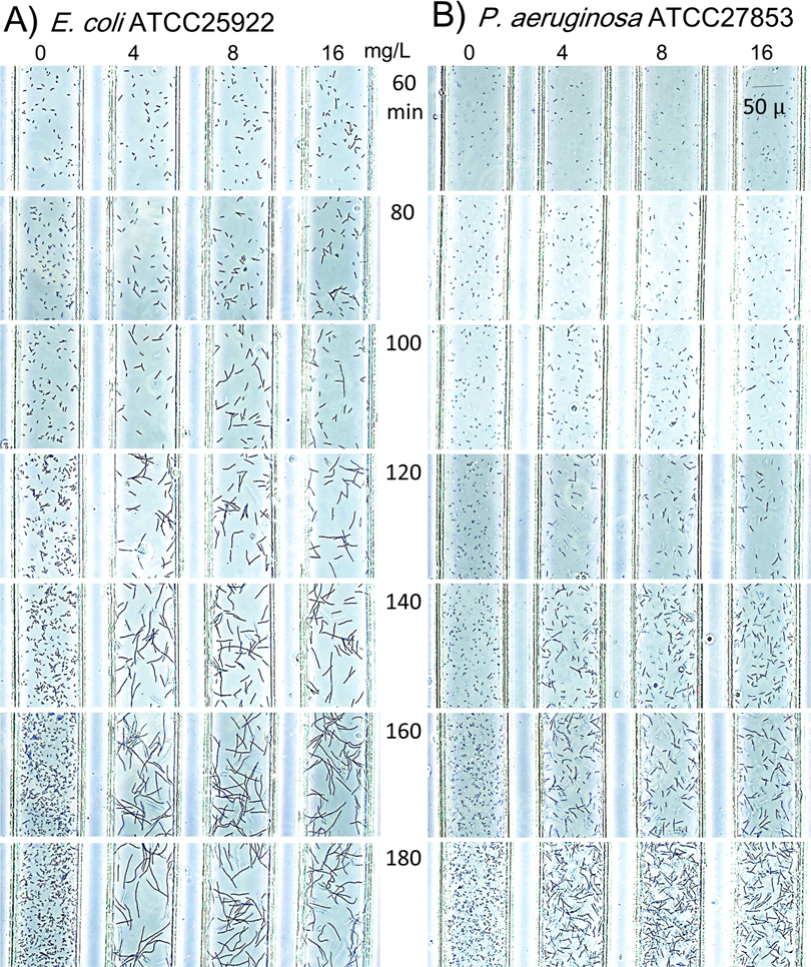
**Time-lapse analysis of A) *Escherichia coli* ATCC25922 and B) *Pseudomonas aeruginosa* ATCC27853 tested by piperacillin using the DSTM method.** Bacterial suspension of McFarland 0.2–0.3 were used. Morphological changes were visible after 80 min in *E*. *coli* and 120 min in *P*. *aeruginosa*.

**Fig 6:**
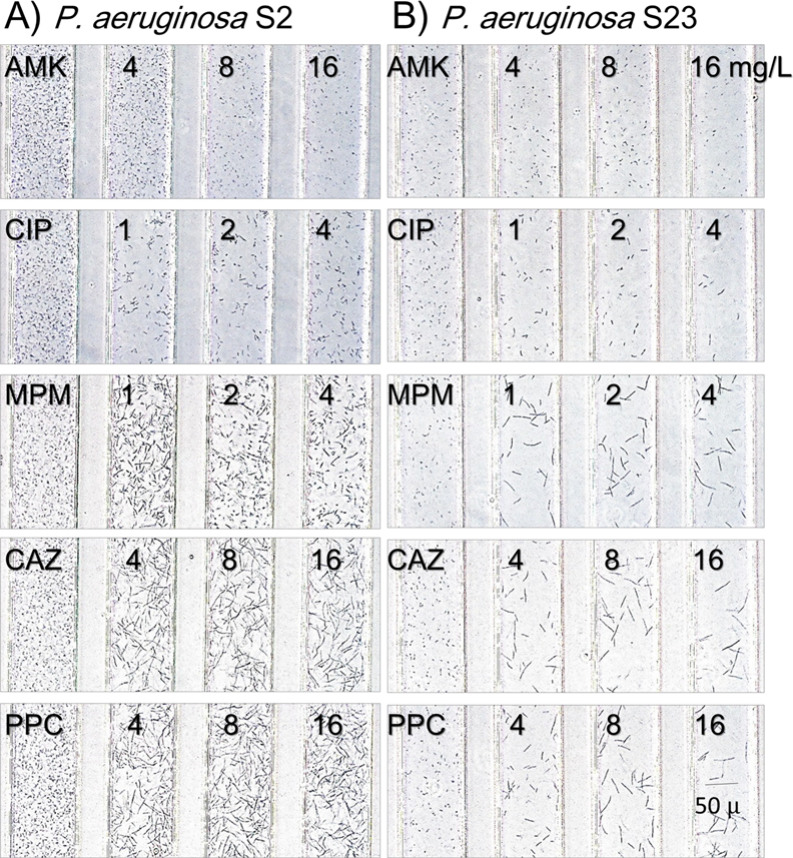
Morphological changes of susceptible strains caused by the five antimicrobial agents. Images were taken after 3 h incubation. A) *Pseudomonas aeruginosa* S2, B) *P*. *aeruginosa* S23. AMK: amikacin, CIP: ciprofloxacin, MPM: meropenem, CAZ: ceftazidime, PPC: piperacillin.

**Fig 7:**
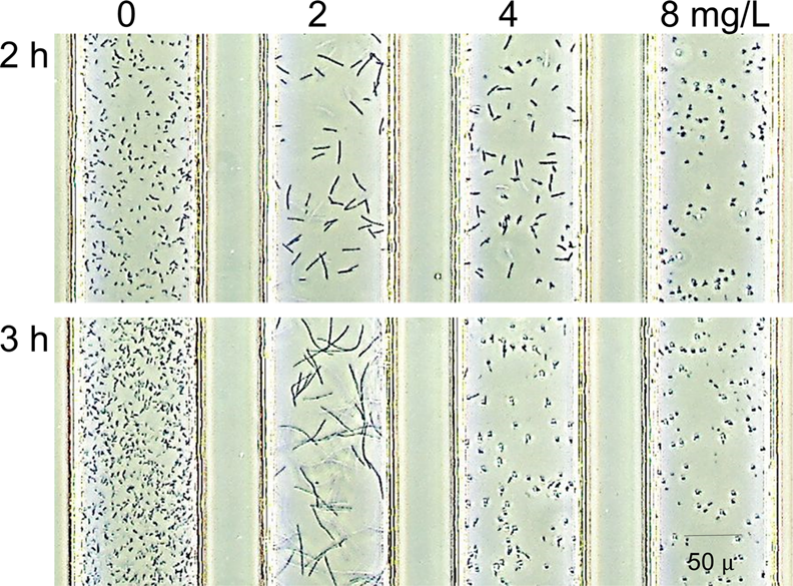
Various morphologies caused by meropenem in a clinical isolate of *Pseudomonas aeruginosa*. Cell elongation was seen at 2 mg/L, bulge formation was observed at 4 mg/L, and spheroplast formation was observed at 4 and 8 mg/L of meropenem using this strain.

*E*. *coli* ATCC25922 and *P*. *aeruginosa* ATCC27853, standard strains for susceptibility testing, were evaluated every 20 min in the DSTM device against PPC for up to 180 min (Fig 5). Bacterial cells became gradually elongated over time. *E*. *coli* grow rapidly, and it is easy to detect morphological changes at 80 min, even though morphological changes were only visible at 120 min for *P*. *aeruginosa*. Fig 6 shows morphological changes in two susceptible strains of *P*. *aeruginosa* caused by the five antimicrobial agents after 3 h incubation. Elongation was visible in the presence of CIP, MPM, CAZ, and PPC, and the levels of changes were increased in this order. Spheroplast and/or bulge formation was observed in the presence of higher concentrations of MPM (Fig 7). On the other hand, there was little difference in the shape of AMK-treated cells, although some of them appeared smaller and displayed a loss of density using microscopy (Fig 6). Morphological changes in the *Pseudomonas aeruginosa* cells in response to these drugs were visible from 2 h of incubation (Fig 5), and susceptibility determinations at 2 h incubation were useful in >90% of strains for these drugs, except for AMK, which caused almost no morphological changes (Fig 6). More than 2.5 h of incubation was needed to easily detect differences in the shapes of drug-treated cells (Fig 5).

### Drug susceptibility criteria in the DSTM method

In comparison with the control, bacterial cell counts were mostly useful as an indicator of drug susceptibility for all drugs tested, and the differences in cell counts were also visible at 3 h, whereas it was sometimes difficult to distinguish differences at 2 h incubation especially in AMK treated channels (Fig 6). The cell counts in drug-treated channels often appeared to increase from the initial image, because *P*. *aeruginosa* cells gradually attached to the surface of the channels as time proceeded. In addition to this, elongated cells tend to lose motility, and sink to the bottom of the fluids (Fig 6), which can be clearly photographed. On the other hand, control cells and resistant cells were very motile. Therefore, the difference in cell counts compared with control in the same view was useful in judging the sensitivity of the test strain.

**Fig 8:**
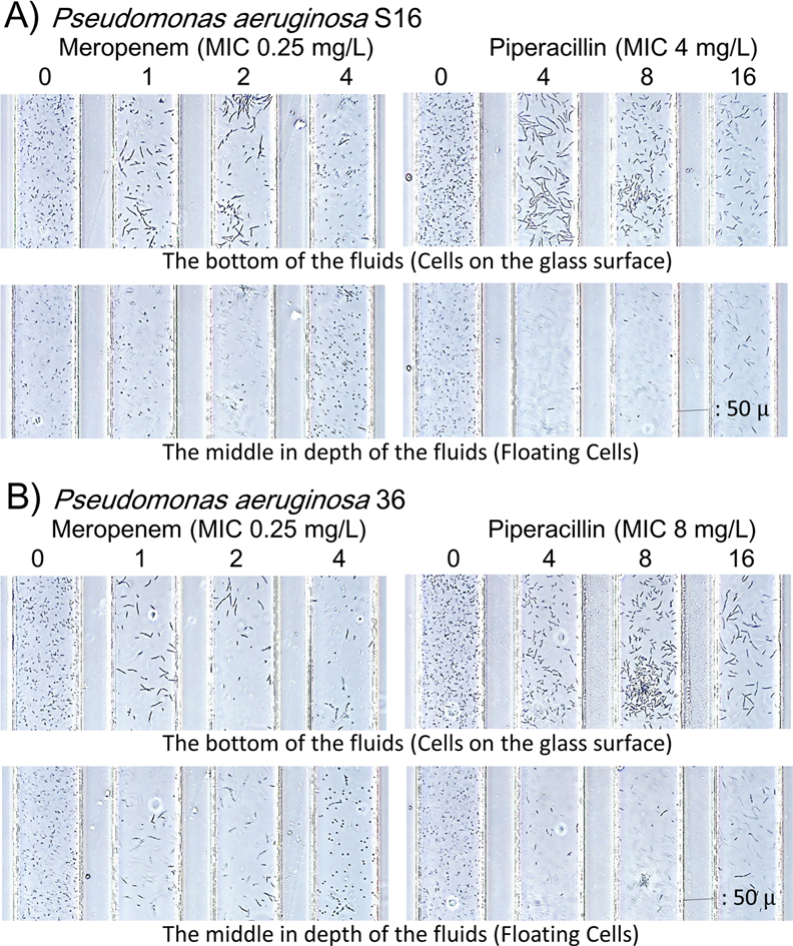
Different image outputs between the bottom and the middle depths of the channels. Images from A) strain S16 and B) strain 36 at 3 h. Elongated cells were usually located on the glass surface, while spheroplast cells were floating in the fluids. The images focused on the glass surface are preferable for analysis using the software.

The depth of channels is 50 μm in the DSTM device. Occasionally, we could see different images using a focused depth (Fig 8). In MPM-treated channels in particular, elongated cells were mostly in the lower images, and easily observed in the picture, although spheroplast cells typically floated in the middle depth images. In PPC-treated channels, elongated cells were mostly observed in the lower images. Images from the bottom of the fluids seemed to be suitable for processing using this type of software. From these images of the 101 strains of *P*. *aeruginosa*, we decided to assess the susceptibility criteria for each drug (Table 1). According to these criteria, MICs were read as the lowest concentration that was judged to be susceptible. The criteria in Table 1 was derived through trial and error to obtain the best correlation between the MICs from the DSTM method and the standard MIC. The bacterium was considered sensitive to drug when the ratio of cell counts versus the control was ≤0.7 for these drugs, except ≤0.6 for PPC. Additionally, reduction of total pixel density was added to AMK susceptibility, as significant morphological changes were not induced with AMK. The level of increased cell length was added to other drug susceptibilities respectively (Table 1). Susceptibilities to CIP were easy to assess by decreases in cell counts and elongated shapes. The difference in cell counts versus the control was also significant in response to MPM, and MPM caused multiple morphological changes (Fig 7) such as elongation at lower concentrations, and bulge or spheroplast formation at higher concentrations. Currently, the software cannot robustly distinguish spheroplast cells from normal cells but it does not prevent susceptibility determination using the software. Although elongation was typical in CAZ- and PPC-susceptible cells, we often observed significant quantities of normal cells that appeared to be resistant to antimicrobial agents among elongated sensitive cells in the β-lactam susceptibility tests. In such cases, we determined it resistant in this method, because resistant cells would overtake sensitive cells and grow completely after overnight incubation using typical methods. This type of resistance was difficult to detect using the software

**Table 1:** Criteria used to denote “susceptible” in the DSTM method using the software.

Drug	Criteria used to denote susceptible (ratio to control)
**Amikacin**	**Cell count ≤0.7 or Pixel density ≤0.7**
**Ciprofloxacin**	**[Cell count ≤0.7] or [Cell count 0.7–0.75 and Average length ≥1.25]**
**Meropenem**	**Cell count ≤0.7 or Average length ≥1.6**
**Ceftazidime**	**[Cell count ≤0.7 except Average length <1.2] or [Average length ≥1.6]**
**Piperacillin**	**[Cell count ≤0.6 except Average length <1.2], [Cell count 0.6–0.7 and Average length ≥1.6] or [Average length ≥2.0]**

Incubation for 3 h appeared sufficient for susceptibility determination in *P*. *aeruginosa* strains using the DSTM method.

### Assessment of accuracy

The reproducibility of the DSTM method was evaluated by testing the standard strain ATCC27853 twelve times using the same cell suspension and different suspensions on the same day, and on different days. Table 2 shows the results obtained using the DSTM method and those obtained using the microbroth dilution method on the same day using the same suspensions. The DSTM method of results was reproducible, although we observed a 4-fold difference from the typical MIC of MPM on one occasion.

**Table 2:** Accuracy of the DSTM method vs. the microbroth dilution method.

Day	1									2	3	4	Distribution
Suspension	A			B			C			D	E	F	
Experiment	1	2	3	4	5	6	7	8	9	10	11	12	
MICs (mg/L) from the DSTM method													
AMK	≤4	≤4	≤4	≤4	≤4	≤4	≤4	≤4	≤4	≤4	≤4	≤4	≤4
CIP	≤1	≤1	≤1	≤1	≤1	≤1	≤1	≤1	≤1	≤1	≤1	≤1	≤1
MPM	≤1	≤1	≤1	≤1	≤1	≤1	≤1	≤1	**2**	≤1	≤1	≤1	≤1–2
CAZ	≤4	≤4	≤4	≤4	≤4	≤4	≤4	≤4	≤4	≤4	≤4	≤4	≤4
PPC	≤4	≤4	≤4	≤4	≤4	≤4	≤4	≤4	≤4	≤4	≤4	≤4	≤4
MICs (mg/L) from the microbroth dilution method													
AMK	2	2	2	4	2	4	2	2	2	4	2	2	2–4
CIP	0.5	0.5	0.5	0.25	0.25	0.25	0.25	0.25	0.5	0.5	0.5	0.5	0.25–0.5
MPM	0.5	0.5	0.5	0.5	0.5	0.5	0.5	0.5	**0.5**	0.25	0.5	0.5	0.25–0.5
CAZ	1	1	1	1	1	1	1	1	1	1	2	1	1–2
PPC	4	4	4	8	4	4	4	4	4	4	4	4	4–8

### Correlation between the DSTM and microbroth dilution methods

**Fig 9:**
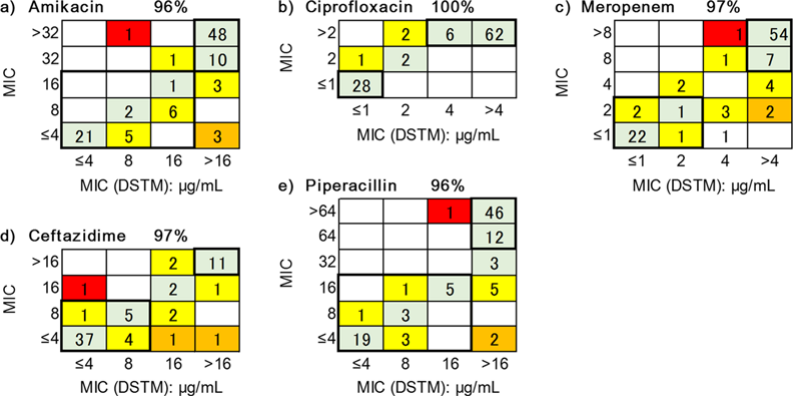
Correlation between MICs from the DSTM method and the microbroth dilution method in *Pseudomonas aeruginosa*. Domains in the bold-lines indicate sensitive or resistant (categorized by the breakpoints of CLSI) using both methods. Green shade, matching; yellow shade, 2-fold difference; orange and red shade, >4-fold difference. Red shade indicates very major errors. The matching ratios (%) shown for each drug contain 2-fold differences.

Determinations of MICs via the DSTM method and the microbroth dilution method were performed on the same day using the same bacterial suspension. When ≥2-fold difference was obtained between the results of the two methods, the strain was re-tested using both methods for confirmation. The determination of MICs after 2 h of incubation was possible for the majority of *P*. *aeruginosa* strains, but it was sometimes difficult to determine the MICs, particularly in response to AMK. One of the strains grew slowly, preventing useful results being obtained after 3 h of incubation using the DSTM method. The correlations of MICs obtained for the remaining 101 strains using the DSTM method after 3 h of incubation with those determined using the microbroth dilution method are shown in Fig 9 for each drug. Although the data were strongly correlated, the MICs obtained using the DSTM method tended to indicate resistance to AMK. Including samples, which had 2-fold difference, the matching rates were 96%, 100%, 97%, 97%, and 96% for AMK, CIP, MPM, CAZ, and PPC, respectively. These included very major errors of 2%, 0%, 2%, 1% and 1%, respectively (Fig 9, Tables B–D in S1 File).

## Discussion

The key to the rapidity of the DSTM method is the introduction of a microscope for susceptibility judgment. Previously, drug susceptibilities were sometimes judged microscopically for *Mycobacterium sp*. on agar medium [55–60]. In addition, various new microscopic methods [42–51, 61, 62] have been recently proposed for ordinary species of gram-negative and gram-positive bacteria. In these methods, only Choi J. et al.[42, 61] described the susceptibility results from *P*. *aeruginosa* obtained over a 3–4 h period. Using a microfluidic agarose channel chip, they determined MICs by analyzing agar-trapped bacterial number and size in response to drugs. Their method is similar to the DSTM method in analyzing not only bacterial number but also bacterial size. However, it is different from the DSTM method in using a unique 96-well plate for the assay. In addition to the complicated process of setting, Choi and colleagues had to take the images one by one at certain time intervals. No other method was available that could obtain susceptibility results in ≤3 h from *P*. *aeruginosa* although many of these rapid methods did use microfluidic chips,[43, 44, 46–49, 62] and were able to determin susceptibility from the growth curves analyzed from time-lapse images. Most notably, Price, C. S. et al.[62] automatically analyzed time-lapse images of the growth of a few drug-treated cells in comparison with control cells of *Staphylococcus aureus*. Using the automated microscopy system, they obtained susceptibility results over a period of 2–4 h, although they could not assay multiple strains simultaneously using their system. Additionally, they required 2 h pre-incubation to obtain logarithmically growing cells, which were applicable for susceptibility testing using the system. This system is also available for rapid identification,[63] and has already been applied in clinical situations.[64]

Drug susceptibility determination of one strain by one expensive system is not efficient, and cost-benefit performance is unlikely to be attained for the majority of hospitals. As an inexpensive, useful solution, we devised microfluidic channels to easily observe simultaneous multiple samples under a microscope. We could easily visualize damaged bacterial cells under a microscope, which were treated with antimicrobials using the DSTM method, compared with control cells. We found that in addition to suppressed growth, morphological changes and a decreased motility (not yet currently available for processing using the software) were also useful as parameters of drug susceptibility in *P*. *aeruginosa* strains. There was no need to perform time-lapse analysis or perform a comparison with the initial image, and we were able to determine the drug susceptibilities of *P*. *aeruginosa* strains to various antimicrobials using only the difference between images for drug-treated channels and control channels at the same time. Results were obtained using this method for *P*. *aeruginosa* within 3 h, and correlated well with the ordinary method (Fig 9).

Before we started this project, we used a microdevice with 17 separate channels to evaluate efflux pump activity in *Escherichia coli*.[39] The device could permit enzyme reactions following short-term incubation, but it was not useful for bacterial cultivation. In addition, PDMS is not hydrophilic, and the introduction of mixtures of bacterial suspensions and drugs into each channel was time consuming. To avoid these limitations, we designed a DSTM device where four channels shared a single inlet hole. Antimicrobials could be fixed in the outlet holes before use, and were simultaneously dissolved with the microbial suspension in each channel. We found that air ingress could completely prevent contamination between channels. Appropriate hydrophobicity of the glass surface in channels blocks flow-back of the suspension, and maintains the independence of each channel. The next requisite was to simultaneously observe the associated channels microscopically. Four channels sharing an inlet hole were gathered in parallel in the observation area near the outlet holes, and each channel was narrowed to 100 μm in width with 50 μm spaces between the channels. When we use ×10 magnification for the objective lens, we could observe up to six channels at the same time microscopically. Additionally, bacterial populations are typically not homogeneous during susceptibility testing, and testing using a small number of cells does not always accurately reflect susceptibility. To evaluate sufficient numbers of cells, we increased the height of the fluids in the DSTM device from 15 μm to 50 μm. By shifting the focus depth, we could further observe various features of damaged cells (Fig 8). Finally, the most important requisite was to support bacterial growth efficiently in the DSTM device. *P*. *aeruginosa* is aerobic, and its growth rate is accelerated by aeration. Most of the air for bacterial growth in the DSTM device appeared to be supplied from the outlet hole near the observation area, which consequently improved growth as the distance between the observation area and the outlet hole decreased. From this point, outlet holes should be positioned at the same distance from the observation area to obtain a similar growth rate in all channels. After trial and error, we optimized the DSTM design as shown in Fig 1. In this method, we can consider the outlet holes in the DSTM device as the wells of a microplate, and channels are prominent structures derived from the wells for easy observation and a simple introduction of bacterial suspensions. As the distance of the channels between the holes and the observation areas decreases, the concentration gradient between the holes and the observation areas also decreases. Bacterial growth is also better in devices with shorter channels because of the good oxygen supply.

The susceptibility results obtained using the DSTM method applied for *P*. *aeruginosa* were correlated with the results obtained using the typical microbroth dilution method, and the agreement rates (≤2-fold difference) were ≥90% for all five agents tested (Fig 9). Reproducibility was also good (Table 2). Although the DSTM method uses a larger inoculum, the short incubation period prevented the increase of the MICs in the large inoculum. However, the MICs of AMK determined using the DSTM method were sometimes 2-fold higher than those determined using the standard method, and those of CAZ and PPC were lower in the DSTM method than for the standard method. In the former situation, the large inoculum size in the DSTM method presumably results in the consumption of antimicrobials surrounding the surface of the bacterial cells. In the latter case, the small number of resistant cells in the population can grow sufficiently after overnight culture using the ordinary method. According to these observations, susceptibility criteria were defined for each antimicrobial to achieve maximum agreement rates using the ordinary method. Therefore, the criterion was different for each drug. To overcome this problem, we had to develop software to analyze the microscopic images for the DSTM method. The software will be supplied together with the DSTM device in the near future.

The DSTM device requires ≤10 min for set-up, and after 3 h of incubation, the microscopic determination of susceptibility of an isolate against five antibiotics can be performed in 10 min using the software. The DSTM method is applicable for susceptibility evaluation to detect resistant strains or to select appropriate drugs. The most significant benefit of the method is its rapidity, and we could obtain a susceptibility result of 99% for *P*. *aeruginosa* in ≤3 h. In this method, changes in cell numbers, shapes and motilities are useful for susceptibility evaluation. Furthermore, software for the automatic analysis of DSTM images will possibly make this method even more rapid and convenient.

**Fig 10:**
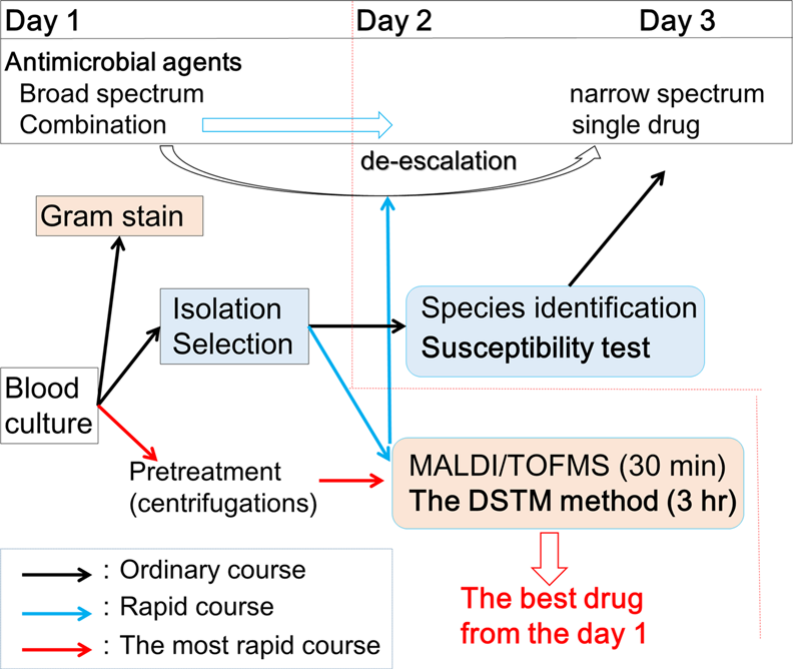
Applicability of the DSTM method in clinical laboratories. When we test positive blood culture, species name and susceptibility data of the causative organism will be available on day 3 using the ordinary method (black arrow course), and available on day 2 using the rapid methods (blue arrow course). Furthermore, those data are available on day 1 by testing blood culture directly after pre-treatment using the rapid methods (red arrow course). Consequently, we can choose the best drug for each patient on day 1, which offers significant clinical advantage.

The need for rapid drug susceptibility testing methods is increasing in situations in which several rapid assays[38, 65–68] have been developed for the identification of strains. However, these assays are not useful for drug susceptibility determination. The DSTM method will be useful in conjunction with these rapid identification methods. We could get susceptibility results of the strain from a blood culture on the same day by testing blood cultures directly after pre-treatment, using the DSTM method (Fig 10). A clinical trial has been started in Japan. The drug combinations used in the DSTM device for testing *P*. *aeruginosa* are also useful for susceptibility testing in other glucose non-fermenters such as *Acinetobacter baumanii*. Furthermore, the drugs can be changed depending on the purpose of the assay. At present, we are expanding the application of the DSTM device to other species such as extended spectrum β-lactamase producing gram-negative bacteria, various gram-negative and gram-positive bacteria, and fungi. When the DSTM technology is put into practical use, bacterial susceptibility testing will become simple and rapid with good cost performance, and omitting the need for expensive equipment.

## Supporting Information

S1 File Materials and Methods. MBL and AAC(6')-Iae Producibility, and Expression Levels of *mexB*, *mexY*, and *oprD* in Each Susceptibility Group of *P*. *aeruginosa* (**Table A**). Comparison of MICs Determined using the DSTM-Method and the Microbroth Dilution Method in Sensitive Strains (**Table B**). Comparison of MICs Determined using the DSTM-Method and the Microbroth Dilution Method in Resistant Strains (**Table C**). Comparison of MICs Determined using the DSTM-Method and the Microbroth Dilution Method in MDR Strains (**Table D**). **References**.
(PDF)
