# Supplementary material for: A Microfluidic Channel Method for Rapid Drug-Susceptibility Testing of Pseudomonas aeruginosa
Source: PLoS One. 2016 Feb 12;11(2):e0148797. doi: 10.1371/journal.pone.0148797 (PMC4752270; doi:10.1371/journal.pone.0148797)
Supplement: S1 File — MBL and AAC(6')-Iae Producibility, and Expression Levels of mexB, mexY, and oprD in Each Susceptibility Group of P. aeruginosa (Table A). Comparison of MICs Determined using the DSTM-Method and the Microbroth Dilution Method in Sensitive Strains (Table B). Comparison of MICs Determined using the DSTM-Method and the Microbroth Dilution Method in Resistant Strains (Table C). Comparison of MICs Determined using the DSTM-Method and the Microbroth Dilution Method in MDR Strains (Table D). References. (PDF) [file pone.0148797.s001.pdf]

Supporting Information

S1 File

A Microfluidic Channel Method for Rapid Drug-Susceptibility Testing of *Pseudomonas aeruginosa*

Yoshimi Matsumoto*, Shouichi Sakakihara, Andrey Grushnikov, Kazuma Kikuchi, Hiroyuki Noji, Akihito Yamaguchi, Ryota Iino, Yasushi Yagi, and Kunihiko Nishino

Corresponding author. E-mail: yoshimi@sanken.osaka-u.ac.jp

This PDF file S1 includes:

Materials and Methods

Table A. **MBL and AAC(6')-Iae Producibility, and Expression Levels of *mexB*, *mexY*, and *oprD* in Each Susceptibility Group of *P. aeruginosa*.**

Table B. Comparison of MICs Determined using the DSTM-Method and the Microbroth Dilution Method in Sensitive Strains.

Table C. Comparison of MICs Determined using the DSTM-Method and the Microbroth Dilution Method in Resistant Strains.

Table D. Comparison of MICs Determined using the DSTM-Method and the Microbroth Dilution Method in MDR Strains.

References

Materials and Methods

Bacterial strains. The same clinically isolated *Pseudomonas aeruginosa* strains in the manuscript were used. *P. aeruginosa* PAO1 was used as a reference strain for qRT-PCR. *P. aeruginosa* IMCJ2.S1 was provided by Dr. T Kirikae and used as a reference strain producing AAC(6')-Iae.

Detection of metallo-β-lactamase (MBL). MICs of imipenem (IPM) were determined in the laboratories of BML, Inc. using conventional agar dilution method with or without 200 mg/L of DPA. A strain exhibiting greater than 4-fold decrease in MICs of IPM in presence of DPA was defined as an MBL producer. MBL producibility was also checked by an immunochromatographic assay kit (Quick chaser IMP: Mizuho Medy Co., Ltd., Japan).

Detection of 6'-N-aminoglycoside acetyltransferase-Iae (AAC(6')-Iae). AAC(6')-Iae producers were identified using an immunochromatographic assay developed by Kitao T, et al.(1) The assay kit was generously provided by Mizuho Medy Co., Ltd. (Tosu, Saga, Japan)

Gene transcription analysis by qRT-PCR. Expression levels of mexB, mexY, and oprD was evaluated by qRT-PCR. Strains were cultured overnight in BBL™ Tripticase™ soy broth (BBL: Becton, Dickinson and Company, Japan) and then inoculated into fresh broth until the culture reached an OD600 of 0.6. Total RNA was isolated using NucleoSpin RNA II kit (Nippon Genetics Co. Ltd, Tokyo, Japan), reverse transcribed using TaqMan reverse transcription reagents, and analyzed using an ABI Prism 7000 Sequence Detection System (Applied Biosystems Japan, Tokyo, Japan) with the KAPATM SYBR Fast qPCR kit (Nippon Genetics Co. Ltd.). The qRT-PCR primer sequences were used as previously described.(2, 3) Samples were run in duplicate. Mean gene expression levels were normalized to those of the ribosomal gene rpsL and were compared after calculating ratios of these levels to those of the reference strain PAO1.

Table A. Expression levels of *mexB*, *mexY*, and *oprD* in each susceptibility group of *Pseudomonas aeruginosa*.

	Expression ratio (PAO1 = 1.0)	Sensitive (26*) N	(%)	Resistant (21*) N	(%)	MDR (54*) N	(%)
mexB	<2	10	(38 %)	8	(38 %)	10	(19 %)
	2 – 4	6	(23 %)	5	(24 %)	15	(28 %)
	4 – 8	7	(27 %)	6	(29 %)	16	(30 %)
	>8	3	(12 %)	2	(10 %)	13	(24 %)
mexY	<2	6	(23 %)	2	(9.5 %)	2	(3.7 %)
	2 – 4	8	(31 %)	1	(4.8 %)	4	(7.4 %)
	4 – 8	7	(27 %)	5	(24 %)	7	(13 %)
	>8	5	(19 %)	13	(62 %)	41	(76 %)
oprD	>0.5	13	(50 %)	6	(29 %)	6	(11 %)
	0.1 – 0.5	1	(3.8 %)	6	(29 %)	9	(17 %)
	0.01 – 0.1	4	(15 %)	1	(4.8 %)	1	(1.9 %)
	<0.01	8	(31 %)	8	(38 %)	38	(70 %)
MBL		0		0		37	(69 %)
AAC(6')-Iae		0		0		33	(61 %)
mexB	>4	10	(38 %)	8	(38 %)	29	(54 %)
mexY	>8	5	(19 %)	13	(62 %)	41	(76 %)
oprD	<0.1	12	(46 %)	9	(43 %)	39	(72 %)

Drug susceptibilities of the clinically isolated 101 strains were determined by the agar dilution method in BML. Sensitive; sensitive to imipenem, ciprofloxacin and amikacin, Resistant; resistant to one or two of before mentioned three drugs, MDR; multidrug-resistant (resistant to all of the three drugs), MBL; metallo-β-lactamase, 6'-N-aminoglycoside acetyltransferase-Iae; AAC(6')-Iae, *; number of strains in each group, N; number of strains

Table B. Comparison of MICs determined using the DSTM-method and the microbroth dilution method in sensitive strains.

Strain	Amikacin		Ciprofloxacin		Meropenem		Ceftazidime		Piperacillin	
	DSTM	CLSI	DSTM	CLSI	DSTM	CLSI	DSTM	CLSI	DSTM	CLSI
ATCC	≤ 4	4	≤ 1	0.5	≤ 1	≤0.25	≤ 4	1	≤ 4	4
S1	≤ 4	4	≤ 1	0.25	≤ 1	1	≤ 4	2	≤ 4	4
S2	≤ 4	2	≤ 1	0.5	≤ 1	0.5	≤ 4	2	8	4
S3	≤ 4	≤ 1	≤ 1	0.5	≤ 1	≤0.25	≤ 4	2	8	4
S4	≤ 4	2	≤ 1	0.5	≤ 1	≤0.25	≤ 4	4	16	16
S5	≤ 4	4	≤ 1	0.25	**4**	**0.5**	≤ 4	2	8	8
S6	8	4	≤ 1	0.25	≤ 1	1	≤ 4	2	8	4
S7	≤ 4	4	≤ 1	0.5	≤ 1	≤0.25	≤ 4	2	≤ 4	8
S8	≤ 4	2	≤ 1	0.5	≤ 1	2	≤ 4	1	≤ 4	4
S10	≤ 4	2	≤ 1	0.25	**2**	**0.5**	≤ 4	2	8	8
S11	**32**	**2**	≤ 1	0.5	≤ 1	1	≤ 4	1	≤ 4	4
S12	≤ 4	4	≤ 1	0.25	≤ 1	≤0.25	≤ 4	2	≤ 4	4
S13	≤ 4	2	≤ 1	0.25	≤ 1	1	≤ 4	1	≤ 4	4
S14	≤ 4	2	≤ 1	0.25	≤ 1	1	≤ 4	4	≤ 4	4
S15	≤ 4	2	≤ 1	0.25	≤ 1	0.5	≤ 4	1	≤ 4	4
S16	≤ 4	2	≤ 1	0.25	≤ 1	0.5	≤ 4	2	≤ 4	4
S17	**8**	**2**	≤ 1	0.25	≤ 1	0.5	≤ 4	2	≤ 4	4
S18	≤ 4	2	≤ 1	0.25	≤ 1	0.5	≤ 4	1	≤ 4	4
S19	≤ 4	≤ 1	≤ 1	0.25	≤ 1	≤0.25	≤ 4	1	≤ 4	1
S20	≤ 4	2	≤ 1	0.25	≤ 1	0.5	≤ 4	1	≤ 4	4
S21	8	4	≤ 1	0.25	≤ 1	0.5	≤ 4	4	≤ 4	4
S22	≤ 4	2	≤ 1	0.25	≤ 1	≤0.25	≤ 4	1	≤ 4	4
S23	≤ 4	2	≤ 1	0.25	≤ 1	≤0.25	≤ 4	1	≤ 4	4
S24	16	8	≤ 1	0.5	≤ 1	≤0.25	≤ 4	1	≤ 4	4
36	≤ 4	1	≤ 1	0.25	≤ 1	0.5	≤ 4	4	8	16
89	≤ 4	4	≤ 1	0.25	**8**	**2**	**8**	**1**	**32**	**4**

ATCC; *Pseudomonas aeruginosa* ATCC27853

Bold letters mean more than 4-fold discrepancy between the MICs obtained by the two methods.

Table C. Comparison of MICs determined using the DSTM-method and the microbroth dilution method in resistant strains.

Strain	Amikacin		Ciprofloxacin		Meropenem		Ceftazidime		Piperacillin	
	DSTM	CLSI	DSTM	CLSI	DSTM	CLSI	DSTM	CLSI	DSTM	CLSI
3	**8**	**1**	≤ 1	2	2	2	4	1	4	4
6	16	8	>4	4	>4	>8	4	4	>16	64
12	>16	> 32	>4	>2	>4	>8	8	4	>16	64
13	>16	> 32	>4	>2	>4	8	4	2	16	16
15	>16	32	>4	4	1	2	4	2	>16	>64
16	16	8	4	>2	>4	>8	4	8	>16	>64
18	**>16**	**4**	2	2	>4	8	4	4	>16	16
22	16	8	>4	>2	>4	>8	>16	>16	>16	>64
23	>16	32	>4	>2	4	2	4	4	16	16
38	>16	> 32	>4	>2	>4	4	4	2	8	8
39	>16	> 32	>4	>2	>4	8	16	8	>16	64
44	>16	> 32	>4	>2	>4	>8	>16	>16	16	16
45	>16	32	>4	>2	>4	>8	>16	>16	16	16
52	>16	> 32	>4	>2	>4	>8	8	8	>16	>64
55	>16	> 32	>4	>2	>4	>8	8	4	>16	>64
56	16	8	>4	>2	4	8	**4**	**16**	>16	>64
60	>16	32	>4	>2	>4	8	**16**	**4**	>16	32
61	>16	16	>4	>2	>4	>8	4	4	>16	16
63	>16	> 32	>4	>2	>4	>8	4	2	>16	>64
66	16	8	>4	>2	>4	4	>16	16	>16	>64
68	>16	4	>4	>2	4	2	4	1	>16	>64
69	>16	> 32	≤ 1	0.25	2	4	4	1	4	4
78	16	16	4	>2	**>4**	**2**	4	2	**>16**	**4**
79	8	8	2	>2	4	2	8	8	>16	16
82	>16	16	4	>2	>4	>8	**>16**	**2**	>16	16
85	8	8	>4	>2	>4	>8	8	4	>16	16
97	>16	32	≤ 1	0.25	>4	8	>16	>16	>16	>64
98	>16	32	>4	>2	>4	4	8	8	>16	64
105	>16	32	>4	>2	>4	>8	8	8	>16	>64
107	4	4	2	2	>4	8	8	8	>16	64

Bold letters mean more than 4-fold discrepancy between the MICs obtained by the two methods.

Table D. Comparison of MICs determined using the DSTM-method and the microbroth dilution method in MDR strains.

Strain	Amikacin		Ciprofloxacin		Meropenem		Ceftazidime		Piperacillin	
	DSTM	CLSI	DSTM	CLSI	DSTM	CLSI	DSTM	CLSI	DSTM	CLSI
1	>16	>32	>4	>2	>4	>8	>16	32	>16	>64
2	>16	32	>4	>2	8	4	>16	16	>16	>64
5	>16	>32	>4	>2	>4	>8	>16	>16	>16	64
7	>16	>32	>4	>2	>4	>8	>16	>16	>16	>64
8	>16	>32	>4	>2	>4	>8	>16	>16	>16	>64
9	>16	>32	>4	>2	>4	>8	>16	>16	>16	>64
10	>16	>32	>4	>2	>4	>8	>16	>16	>16	>64
14	>16	>32	>4	>2	>4	>8	16	>16	>16	>64
19	>16	>32	>4	>2	>4	>8	>16	>16	>16	>64
20	>16	>32	>4	>2	>4	>8	>16	>16	>16	>64
21	>16	>32	>4	>2	>4	>8	16	8	>16	>64
24	>16	>32	>4	>2	>4	>8	16	>16	>16	>64
25	>16	>32	>4	>2	>4	>8	>16	>16	>16	>64
29	>16	>32	>4	>2	>4	>8	>16	>16	>16	>64
30	>16	>32	>4	>2	>4	>8	>16	>16	>16	>64
31	>16	>32	>4	>2	>4	>8	>16	>16	>16	>64
32	>16	>32	4	>2	>4	>8	>16	>16	>16	64
33	>16	32	>4	>2	>4	>8	>16	>16	>16	>64
40	>16	16	>4	>2	>4	>8	>16	>16	>16	>64
41	>16	>32	>4	>2	>4	>8	>16	>16	>16	>64
42	>16	>32	>4	>2	>4	>8	>16	>16	>16	>64
46	>16	>32	>4	>2	>4	>8	>16	>16	>16	32
50	>16	>32	>4	>2	>4	>8	>16	>16	>16	>64
54	>16	>32	2	>2	>4	>8	>16	>16	>16	>64
57	>16	>32	>4	>2	>4	>8	>16	>16	>16	>64
62	>16	>32	>4	>2	>4	8	16	16	>16	64
67	>16	>32	>4	>2	>4	>8	>16	>16	>16	>64
71	16	32	>4	>2	>4	>8	>16	>16	>16	64
72	>16	>32	>4	>2	>4	>8	>16	>16	>16	64
74	>16	>32	>4	>2	>4	>8	>16	>16	**16**	**>64**
75	>16	>32	>4	>2	>4	>8	>16	>16	>16	>64
83	>16	32	>4	>2	>4	>8	16	16	>16	>64
86	>16	>32	>4	>2	>4	>8	>16	>16	>16	>64

87	>16	>32	>4	>2	>4	>8	>16	>16	>16	>64
88	>16	>32	>4	>2	>4	>8	>16	>16	>16	>64
91	>16	>32	>4	>2	2	4	>16	>16	>16	>64
92	>16	>32	>4	>2	>4	>8	>16	>16	>16	>64
93	>16	>32	>4	>2	>4	>8	>16	>16	>16	64
94	**8**	**>32**	>4	>2	>4	>8	>16	>16	>16	>64
95	>16	>32	>4	>2	>4	>8	>16	>16	>16	>64
96	>16	>32	>4	>2	**4**	**>8**	>16	>16	>16	32
100	>16	>32	>4	>2	>4	>8	>16	>16	>16	>64
101	>16	>32	>4	>2	>4	>8	>16	>16	>16	>64
103	>16	>32	>4	>2	>4	>8	>16	>16	>16	>64
106	>16	>32	>4	>2	>4	>8	>16	>16	>16	64

Bold letters mean more than 4-fold discrepancy between the MICs obtained by the two methods.

References

1. **Kitao T, Miyoshi-Akiyama T, Shimada K, Tanaka M, Narahara K, Saito N, Kirikae T.** 2010. Development of an immunochromatographic assay for the rapid detection of AAC(6')-Iae-producing multidrug-resistant Pseudomonas aeruginosa. J Antimicrob Chemother **65:**1382-1386.
2. **Yoneda K, Chikumi H, Murata T, Gotoh N, Yamamoto H, Fujiwara H, Nishino T, Shimizu E.** 2005. Measurement of Pseudomonas aeruginosa multidrug efflux pumps by quantitative real-time polymerase chain reaction. Fems Microbiology Letters **243:**125-131.
3. **Dumas JL, van Delden C, Perron K, Kohler T.** 2006. Analysis of antibiotic resistance gene expression in Pseudomonas aeruginosa by quantitative real-time-PCR. FEMS Microbiol Lett **254:**217-225.
